# Compressive properties of commercially available polyurethane foams as mechanical models for osteoporotic human cancellous bone

**DOI:** 10.1186/1471-2474-9-137

**Published:** 2008-10-09

**Authors:** Purvi SD Patel, Duncan ET Shepherd, David WL Hukins

**Affiliations:** 1 School of Mechanical Engineering, University of Birmingham, Edgbaston, Birmingham, B15 2TT, UK

## Abstract

**Background:**

Polyurethane (PU) foam is widely used as a model for cancellous bone. The higher density foams are used as standard biomechanical test materials, but none of the low density PU foams are universally accepted as models for osteoporotic (OP) bone. The aim of this study was to determine whether low density PU foam might be suitable for mimicking human OP cancellous bone.

**Methods:**

Quasi-static compression tests were performed on PU foam cylinders of different lengths (3.9 and 7.7 mm) and of different densities (0.09, 0.16 and 0.32 g.cm^-3^), to determine the Young's modulus, yield strength and energy absorbed to yield.

**Results:**

Young's modulus values were 0.08–0.93 MPa for the 0.09 g.cm^-3 ^foam and from 15.1–151.4 MPa for the 0.16 and 0.32 g.cm^-3 ^foam. Yield strength values were 0.01–0.07 MPa for the 0.09 g.cm^-3 ^foam and from 0.9–4.5 MPa for the 0.16 and 0.32 g.cm^-3 ^foam. The energy absorbed to yield was found to be negligible for all foam cylinders.

**Conclusion:**

Based on these results, it is concluded that 0.16 g.cm^-3 ^PU foam may prove to be suitable as an OP cancellous bone model when fracture stress, but not energy dissipation, is of concern.

## Background

Synthetic bone test specimens are often used in favour of cadaveric specimens, because of their low variance in material properties and availability (when compared to cadaveric specimens), and for the uncontaminated and clean test environment that they provide. Rigid, closed cell polyurethane (PU) foams, with densities typically ranging from 0.16–0.64 g.cm^-3^, are widely used as standard test materials for mimicking human cancellous bone [1]. The PU foam is available in blocks, which have been used to investigate fixation of bone screws [2,3], and will be used in this form for the current study. PU foam is also used as a cancellous core material in whole bone models, with an outer coating to feature cortical bone; these models have been used to investigate devices such as intramedullary nails [4]. The mechanical properties of the whole bone models have been compared with those of natural bone [5].

Little work has been carried out on synthetic materials that might mimic human osteoporotic (OP) cancellous bone. Osteoporosis is a bone disease in which bone resorption exceeds bone deposition, resulting in bone loss [6]. Various open cell rigid PU foams are available for use as OP bone models because of their low material densities (typically around 0.09 g.cm^-3^) [7]. However, a literature search has revealed that hardly any studies exist to determine whether PU foam may be a valid test material for OP cancellous bone. Johnson and Keller [8] reported the mechanical properties of two open cell rigid PU foams, with densities of 0.09 g.cm^-3 ^and 0.12 g.cm^-3^, as models for synthetic thoracic vertebrae. They concluded that the open cell foam provided an alternative for static or fatigue studies of human vertebrae, suggesting that future work could involve various porosity foams to simulate different degrees of OP degeneration [8]. Szivek et al. [9,10] measured the elastic modulus, yield and compressive strength of different, closed cell, PU foam compositions (prepared during their study), which provided reproducible mechanical properties falling within a range of cancellous bone properties from various types of patients. However, the study was not disease-specific when comparing the mechanical properties of the PU foam formulations with the published data. Furthermore, it is not always practical to formulate particular compositions of PU foam, given that there are several commercially available PU foams. Other studies [11-13] have examined PU foams under compression, shear and fatigue, for their use as a cancellous bone analogue material. But none of these studies have specifically characterised PU foam as an OP cancellous bone model, by comparison of the relevant data with OP bone properties.

In this study, the aim was to determine whether any low density PU foam (i.e. open cell or closed cell) might be suitable as a mechanical model for human OP bone. Suitability was determined by measuring the Young's modulus, yield strength and energy absorbed to yield for three PU foams and directly comparing them with the corresponding values obtained from a study of human OP cancellous bone [14]. The determination of such mechanical properties may help selection of the relevant PU foams as an OP cancellous bone model in other studies, for example, in the mechanical evaluation of implant performance [15].

## Methods

### 2.1 PU Foam Samples

PU foams, of three different densities, were used in this study. Closed cell PU foam of density 0.16 g.cm^-3 ^and 0.32 g.cm^-3 ^(American Society for Testing and Materials, ASTM, Grade 10 and Grade 20) [1] was used to model low and medium density cancellous bone respectively. Open cell rigid foam of density 0.09 g.cm^-3 ^was used to model very low density cancellous bone. All PU foams were purchased in block form, with dimensions 130 × 180 × 40 mm, from Sawbones^® ^Europe AB, Malmö, Sweden. The foam densities were supplied by Sawbones^® ^Europe AB.

Using a sharpened tube, six cylindrical cores of 9 mm diameter were drilled from each of the three different density PU foam blocks. The cores were taken using the method described by Li and Aspden [14], in which the cylindrical axis of the core was roughly perpendicular to the surface of the PU block (this is the preferred orientation of the "trabeculae"). The exact diameter of the PU cylinders was determined as an average of four measurements; this was necessary to account for the inhomogeneity of the 0.09 g.cm^-3 ^open cell PU foam in particular.

For this study, two different cylinder lengths were chosen to test for any buckling or shape effects. A cylinder, of length of 7.7 ± 0.2 mm, was chosen so that results could be compared with those from a published study of human OP cancellous bone [14]. In order to investigate the effect of specimen dimensions, a cylinder, of length 3.9 ± 0.1 mm, was also investigated. This length was obtained from a standard for testing rubbers [16]. The reason for choosing this standard was to ensure that the specimens did not bulge during compression; rubbers have a Poisson's value of about 0.5 and so maintain an almost constant volume during compression; as a result, they bulge more than most other materials [17,18]. Dimensions were measured with digital vernier callipers (Fisher Scientific UK Ltd., Leicestershire).

Six cylinders were prepared for each cylinder length and each density of PU foam block. The required cylinder length was achieved by either using a small pair of scissors, for the 0.09 g.cm^-3 ^PU foam, or by rubbing the PU foam cylinder on a sheet of sandpaper (medium grade M2, SupaDec, RS Components Ltd., Northamptonshire, UK), for the 0.16 g.cm^-3 ^and 0.32 g.cm^-3 ^PU foams.

### 2.2 Mechanical Testing

Quasi-static unconstrained compression tests were conducted using an ELF3200 (for the lowest density foam) or an ELF3300 (for other PU foams) materials testing machine (Bose Corporation, ElectroForce Systems Group, Minnetonka, MN, U.S.A.). The ELF3200 testing machine is fitted with a load cell of full scale 225 N (maximum error 0.21% of the full scale) and a displacement transducer with full scale 6.5 mm (maximum error 0.49% of the full scale). The ELF3300 testing machine is fitted with a load cell of full scale 5100 N (maximum error 0.1% of the full scale) and a displacement transducer with full scale 12.7 mm (maximum error 0.28% of the full scale). The manufacturer's tolerances on the hole alignments are ± 0.1 mm.

The lowest density foam was tested using a different machine, with a lower capacity load cell, because of its greater compliance and lower strength. All tests were video-recorded using a video camera (Sony Handycam DCR-DVD404E, Sony Corporation, Japan). No preload or preconditioning was applied to the specimens, which were compressed between two acetal plates (thickness 15 mm). For the 3.9 mm and 7.7 mm cylinder lengths, tests were performed under displacement control at a rate of 0.013 mm.s^-1 ^and 0.026 mm.s^-1 ^respectively, both of which are equivalent to a strain rate of 0.0033 s^-1 ^[14]. Inspection of video recordings showed a repetitive cycle of trabeculae fracture and consolidation (particularly for the 0.09 g.cm^-3 ^PU foam). All test cylinders experienced loads less than the critical load required for Euler buckling and no such buckling was observed in the video images. For each compression test, the engineering stress was calculated by dividing the load recorded at each data point by the original cross-sectional area of the PU foam cylinder, whilst the engineering strain was calculated by dividing the displacement of the machine actuator head (at each data point) by the original height of the PU foam cylinder [19]. A fifth-order polynomial was fitted to the stress-strain curves. The material properties determined were the Young's modulus, the yield strength, and the energy absorbed up to the yield point. A general expression for Young's modulus was found by differentiating the polynomial equation of the engineering stress-strain curve with respect to strain. This expression for Young's modulus was then plotted against strain and the Young's modulus was determined as the maximum value on the curve. It was necessary to determine the Young's modulus in this way because the stress-strain curves were non-linear. The yield strength was determined by the method described by Li and Aspden [14]; i.e. it was determined as the stress at which the Young's modulus had reduced by 3% from its maximum value. The energy absorbed to yield was calculated by integrating the polynomial equation of the engineering stress-strain curve between the limits of zero and the strain point at which the yield strength was determined.

### 2.3 Statistical Analysis

Statistical comparisons were made using MINITAB^® ^Release 14.1 Statistical Software (Minitab Inc., Pennsylvania, USA). Normality of the distributions was assessed using the Anderson-Darling test. Data were compared using the two-sample t-test (normally distributed data) or the Mann-Whitney test (non-parametric data), with the significance level set at 0.05.

## Results

**Figure 1:**
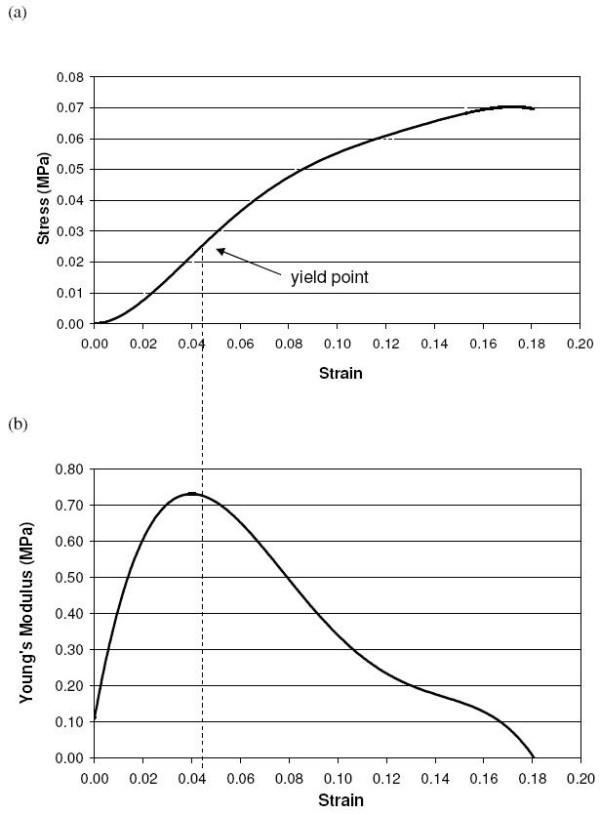
**Stress-strain and Young's modulus-strain curves**. (a) Stress-strain curve from a 7.7 mm length sample of open cell PU foam (0.09 g.cm^-3^) used to model very low density human cancellous bone and (b) the Young's modulus determined from it as the gradient of the curve. The yield point is defined by the point at which the Young's modulus decreases by 3% from its maximum value. The area under the stress-strain curve up to the yield point is defined as the energy absorbed to yield.

Fig. 1a shows a stress-strain curve for a sample of low density PU foam that was tested to failure. A general expression for the Young's modulus of the material is given by the gradient of the curve. Fig. 1b shows the curve obtained if the Young's modulus expression is plotted as a function of strain. The Young's modulus is taken as the maximum value in Fig. 1b. The yield point is defined as the stress at the end of the peak region, when the Young's modulus reduces by 3%. Fig. 1a and 1b have the same strain axes to allow for easy comparison. The curves are typical of those obtained in this study. The energy absorbed to yield is the area under the stress-strain curve up to the yield point.

Table 1 summarises the differences in values for Young's modulus, yield strength and energy absorbed to yield between the 3.9 mm and 7.7 mm length PU foam cylinders. Significant differences were detected in the Young's modulus between 3.9 mm and 7.7 mm length PU foam cylinders for all three PU foam densities (p < 0.05). No significant differences were detected in the yield strength between 3.9 mm and 7.7 mm length PU foam cylinders for all three PU foam densities. For the energy absorbed to yield, significant differences (p < 0.05) were detected between 3.9 mm and 7.7 mm length PU foam cylinders for 0.16 g.cm^-3 ^and 0.32 g.cm^-3 ^PU foam, but not for the 0.09 g.cm^-3 ^PU foam.

**Table 1:** Young's modulus *(E)*, Yield strength and Energy absorbed to yield for PU foam cylinders under compression

**9 mm Diameter PU Foam Cylinder**	**Mean *E *(MPa)**	**Median *E *(MPa)**	**Mean Yield Strength (MPa)**	**Median Yield Strength (MPa)**	**Mean Energy Absorbed to Yield (kJ.m^-3^)**	**Median Energy Absorbed to Yield (kJ.m^-3^)**
0.09 g.cm^-3 ^density foam						
3.9 mm length	0.3 (0.2)	0.3	0.02 (0.01)	0.02	0.001 (0.001)	0.001
7.7 mm length	0.7 (0.2)	0.7	0.04 (0.02)	0.03	0.001 (0.001)	0.001

0.16 g.cm^-3 ^density foam						
3.9 mm length	19 (3)	19	1.0 (0.1)	1.0	0.03 (0.01)	0.03
7.7 mm length	41 (3)	42	1.1 (0.1)	1.1	0.01 (0.003)	0.01

0.32 g.cm^-3 ^density foam						
3.9 mm length	66 (13)	64	3.6 (0.5)	3.6	0.10 (0.05)	0.08
7.7 mm length	145 (6)	146	3.3 (0.9)	3.7	0.03 (0.01)	0.03

The figure in brackets following the mean is the standard deviation (SD).

Table 2 summarises the median values for Young's modulus, yield strength and energy absorbed to yield, found from Li and Aspden's study that investigated the mechanical properties of human OP bone [14]. A direct comparison can be made between the values in Table 2 and the corresponding mechanical property values, for the 7.7 mm PU cylinder length, in Table 1. Table 2 includes the ranges of the 5% to 95% confidence limits from Li & Aspden's study; the ranges were extracted from box plots and are only approximate values.

**Table 2:** Summary of mechanical properties obtained from Li & Aspden's study

**Material Property**	**OP Bone**	**Normal Bone**
*E *(MPa)	247	310
	50 – 410	40 – 460

Yield strength (MPa)	2.5	3.3
	0.6 – 5.8	0.4 – 9.0

Energy absorbed to yield (kJ.m^-3^)	16.3	21.8
	2 – 52	2 – 90

Median values and approximate ranges of the 5% – 95% confidence limits from Li & Aspden's study on the mechanical properties of human cancellous bone specimens (diameter: 9 mm, mean cylinder length: 7.7 mm) from OP femoral heads [14].

## Discussion

The purpose of this work was to determine whether any low density PU foam (open cell or closed cell) might be suitable as a mechanical model for human OP cancellous bone. To the authors' knowledge, this is the only paper that has compared the mechanical properties of PU foams with results from bone [14] using exactly the same methods. The study results provide evidence that at least one out of the three foams tested can be a potential model for OP bone. The results for each density of PU foam are discussed in the following paragraphs.

The 0.09 g.cm^-3 ^PU foam, used to model very low density bone in this study, is much weaker than the OP bone investigated by Li and Aspden [14]. Tables 1 and 2 show that values of Young's modulus, yield strength and energy absorbed to yield, for the 0.09 g.cm^-3 ^PU foam, are below the range of Li and Aspden's results. These findings may highlight a difficulty in using open cell PU foam to model OP cancellous bone. The problems associated with modelling OP bone are discussed later.

For the 0.16 g.cm^-3 ^and 0.32 g.cm^-3 ^PU foam used in this study, the range of the Young's modulus and yield strength was 15.1–151.4 MPa (Young's modulus) and 0.9–4.5 MPa (yield strength). The literature has reported the Young's modulus of human cancellous bone to vary within the range of 1.1–9800 MPa [20-22], and includes human cancellous bone located across the tibia, vertebral bodies and humerus, whilst the yield strength is reported to differ within the range of 0.6–17.5 MPa [23,24], accounting for cancellous bone within the vertebra, tibia and femur. Results for the 0.16 g.cm^-3 ^and 0.32 g.cm^-3 ^PU foam used in this study are within these ranges quoted above; this agreement is to be expected because foams with this density are intended to meet the ASTM standard [1]. However, use of 0.32 g.cm^-3 ^PU foam as a "normal" bone model can be justified to a greater extent than the 0.16 g.cm^-3 ^PU foam; the 0.32 g.cm^-3 ^PU foam gave similar values for Young's modulus and yield strength between this study and Li and Aspden's work on normal bone.

It is difficult to categorise the 0.16 g.cm^-3 ^PU foam as a "normal" or OP bone model. Young's modulus values for the 0.16 g.cm^-3 ^foam are close to the 5% confidence limits of 40 and 50 MPa for normal and OP bone respectively [14]. In addition, yield strength values for the 0.16 g.cm^-3 ^foam are close to the 5% confidence limits of 0.4 and 0.6 MPa for normal and OP bone respectively [14]. These findings suggest that the 0.16 g.cm^-3 ^PU foam may prove suitable as an OP bone model for mechanical testing that is concerned with fracture stress.

Previous studies have concentrated on either open or closed cell PU foams; here we consider both as possible models for OP bone. Open cell and closed cell PU foams have been reported to exhibit different responses to mechanical loads [8]. Open cell foams are favoured for their compressive fatigue behaviour, where the localised single-cell crush band has been found to be more characteristic of cancellous bone, unlike the expandable crush zone found in closed cell foam under the same strain [25,26]. Closed cell foam has been found to exhibit similar static mechanical properties to human cancellous bone, but different characteristics to human bone in fatigue [12], thus supporting the use of 0.16 g.cm^-3 ^PU foam as an OP bone model in fracture studies.

For all the PU foams of different lengths and densities used in this study, the energy absorbed to yield was found to be negligible. This would indicate that PU foam has a more brittle nature compared to human bone. One theory [27] suggests a 'modular' elongation mechanism for the toughness of natural composites such as bone, whereby the domains within a single molecule unfold (or loops open) upon pushing or pulling, so that "sacrificial bonds" are broken before a strong bond is broken (if the force is large enough). Such behaviour cannot be exhibited in a homogeneous material like PU foam. Thus, PU foam may not be a suitable model when energy dissipation (such as in fatigue) is of concern.

The results in this paper suggest that it is difficult to find a synthetic material to mimic the properties of OP bone. In part this is due to the wide spread of results that have been published for real normal and OP bone [14]. Table 2 shows that the yield strength and the energy absorbed to yield are similar for the OP and normal bone. A possible explanation is that normal bone shows considerable individual variability so that when bone tissue is lost, as a result of OP, from some individuals the resulting tissue has properties that resemble those of normal bone from other individuals.

Two different PU cylinder lengths were chosen to determine whether specimen dimensions would affect the results. Significant differences were found in the Young's modulus and energy absorbed to yield (except for the 0.09 g.cm^-3 ^PU foam) between the two PU foam cylinder lengths. This result is consistent with the findings of Keaveny et al. [28] who found a weak dependence between Young's modulus and specimen aspect ratio for cylindrical specimens of cancellous bone. The response of a cellular solid to compression is not simple. Video recordings showed that deformation of the open-cell foams involved bending and buckling of the PU "struts"; failure involved fracture and consolidation. A similar structural response to compression has been observed in the trabeculae of cancellous bone [29]. This complicated response may be implicated in the dependence of the results on specimen geometry. However, the most important conclusion is that any comparison of results from PU foam and bone should be for results obtained from specimens with comparable dimensions.

The mechanical properties of the PU foams used in this study have been derived from a single strain rate, in order to compare the results with those published for cancellous bone [14]. A useful future study would be to test the mechanical properties of the PU foams, considered in this study, when they are subjected to higher strain rates and then to compare the data with mechanical properties of cancellous bone tested at high strain rates. Any similarities found between the mechanical properties for PU foam and cancellous bone would further strengthen the case for using PU foams as a human cancellous bone model.

## Conclusion

PU foam of density 0.16 g.cm^-3 ^may prove suitable as an OP cancellous bone model when fracture stress, but not energy dissipation, is of concern. The 0.16 g.cm^-3 ^PU foam is a good alternative for in-vitro testing because it has compressive Young's modulus and yield strength values similar to OP bone that has also been tested in compression. It has not been possible to characterise the foam through other forms of testing due to the lack of appropriate data to compare our study results with.

## Competing interests

The authors declare that they have no competing interests.

## Authors' contributions

PSDP considered the study, carried out the experimental work, data analysis and drafted the manuscript. DETS participated in the study design, assisted with data interpretation and helped to draft the manuscript. DWLH participated in the study design, assisted with data interpretation and critically revised the manuscript. All authors read and approved the final manuscript.

## Pre-publication history

The pre-publication history for this paper can be accessed here:


